# A methodology framework for bipartite network modeling

**DOI:** 10.1007/s41109-023-00533-y

**Published:** 2023-01-17

**Authors:** Chin Ying Liew, Jane Labadin, Woon Chee Kok, Monday Okpoto Eze

**Affiliations:** 1 Mathematical Sciences Studies, College of Computing, Informatics and Media, Universiti Teknologi MARA, Sarawak Branch, 94300 Kota Samarahan, Sarawak, Malaysia; 2 Faculty of Computer Science and Information Technology, Universiti Malaysia Sarawak, 94300 Kota Samarahan, Sarawak, Malaysia; 3 Department of Computer Science, Babcock University, Ilishan-Remo, Ogun State Nigeria

**Keywords:** Graph theory, Individual-based modeling, Complex network, Habitat suitability, Epidemiology, Disease modeling, Dengue, Irrawaddy dolphin, Heterogenous

## Abstract

The graph-theoretic based studies employing bipartite network approach mostly focus on surveying the statistical properties of the structure and behavior of the network systems under the domain of complex network analysis. They aim to provide the big-picture-view insights of a networked system by looking into the dynamic interaction and relationship among the vertices. Nonetheless, incorporating the features of individual vertex and capturing the dynamic interaction of the heterogeneous local rules governing each of them in the studies is lacking. The methodology in achieving this could hardly be found. Consequently, this study intends to propose a methodology framework that considers the influence of heterogeneous features of each node to the overall network behavior in modeling real-world bipartite network system. The proposed framework consists of three main stages with principal processes detailed in each stage, and three libraries of techniques to guide the modeling activities. It is iterative and process-oriented in nature and allows future network expansion. Two case studies from the domain of communicable disease in epidemiology and habitat suitability in ecology employing this framework are also presented. The results obtained suggest that the methodology could serve as a generic framework in advancing the current state of the art of bipartite network approach.

**Graphical Abstract:**

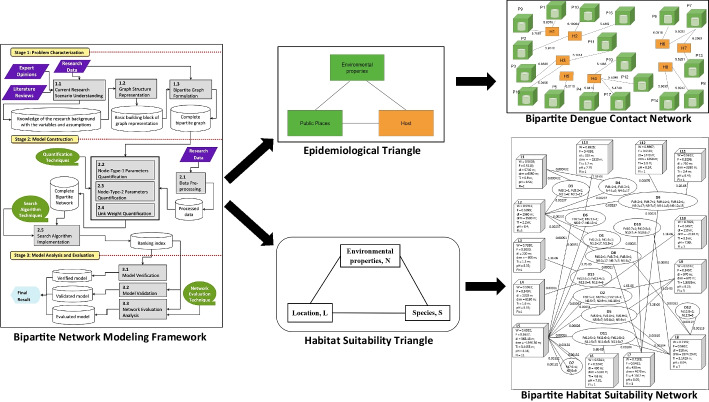

## Introduction

The bipartite network approach applies network theory that has its basis in graph theory (Harary 1969). This graph-theoretic network approach commonly focuses on the properties, the structural dynamics, and the relationship between the structure and function of real-world networks like social networks, transportation systems, collaboration networks, epidemiology and the Web and Internet structures which are regarded as emergent fields of network science by Barabási (2013). A bipartite network consists of nodes of two different natures with links joining only between unlike nodes. It is also referred to as an affiliation or two-mode network (Kevork and Kauermann 2022). The heterogeneous nature of the bipartite network makes it a realistic model of the real-world system and applicable across a wide range of research fields, particularly in the studies related to science and technology (Valejo et al. 2021). It is commented as capable of providing insightful representation from mutualistic networks in ecology to trade networks in the economy (Saracco et al. 2015).

In the well-cited review paper by Newman (2003) on the structure and functions of complex networks, a bipartite network is regarded as both a preference network under the category of information or knowledge network and a type of network under the social network category among the four network categories given. Most of the studies that apply the bipartite graph or bipartite network approach focus on the statistical properties of the structure and behavior of these networked systems under the domain of complex network analysis. The emphasis is to delve into the properties of networks that discusses features like, but not limited to, the small-world effect, transitivity or clustering, degree distribution of the vertices in the network, characteristics of community within a network, resilience of a network, assortativity of the connection between vertices, network clustering that considers the density of edges among vertices and groups with different clustering structure, and navigation within a network (Baumgartner 2020; Derudder 2021; Ducruet and Beauguitte 2014; Kevork and Kauermann 2022).

Complex network analysis has been employed in surveying the relationship between the two types of nodes like different aspects of epidemiological modeling on complex networks (Jin et al. 2016; Zhao et al. 2021), microbes-compound metabolic network (Zhang and Deng 2021), user-object bipartite network in abstracting the selection pattern of web objects (Chandra et al. 2017), the relationship between cyberspace and physical space regarding a grid cyber-physical systems (Huang et al. 2018), hash-tags and users in studying the complex interactions between the semantic content of a debate and the users’ interest in the Twitter activity (Gargiulo et al. 2015), and ecological bipartite networks of biotic interaction types within ecological communities (Kaszewska-Gilas et al. 2021; Poisot et al. 2015). Apart from that, the bipartite network approach has been widely applied in the studies of social sciences or social networks. This includes the studies of disease transmission networks (Büttner and Krieter 2020; Hernándex and Risau-Gusman 2013; Rafo et al. 2021), biological system networks (Baumgartner 2020), food-web networks (Michalko et al. 2021), ecological network (Elliott et al. 2021), cognitive network (Vitevitch et al. 2021), and governance-leadership relationship in a development policy network (Rudnick et al. 2019).

These studies show that the focus in typical complex network research is on the big-picture-view of the networked system and observation of the interaction or relationship between the vertices of the network. Incorporating the features of individual nodes (which are the local rules governing the individual vertices) and capturing the dynamic interaction of the heterogeneous characteristics of the individual node in a network is scarce. Studies on how these could be done are lacking in the above complex network research. As Newman (2003) has pointed out, predicting the system behavior based on the measured structural properties and local rules governing individual vertices is still in its infancy. As a result, the methodology in bipartite network modeling that incorporates unique features of every individual node in a network is also lacking.

Typical modeling processes include understanding or formalizing the research problem to confirm the feasibility of employing an approach in modeling the studied problem before determining the potential variables and assumptions. This is followed by formulation of the intended model incorporating the variables, utilizing an approach that usually comprises iterative processes. Lastly is the process of evaluating the model formulated to validate its usefulness in achieving the intention it is developed. In studies utilizing a novel modeling approach, the last stage usually requires verifying the processes implemented in formulating the model comply with the standard practices of a specific research community.

Studies employing the graph-theoretic network approach predominantly focus on the network data analyses, which are statistically based. The methodology generally includes formalizing the research problem, setting up the bipartite graph by defining the bipartite nodes and the existence of edges between the unlike node types, abstracting the real-world system and formulating it into a bipartite network model, performing analyses onto the network which focus on network structure, validating the bipartite network model and concluding the real-world system based on the findings obtained from the network analyses (Derudder 2021; Kaszewska-Gilas et al. 2021; Kevork and Kauermann 2022). In brief, the primary steps in typical network approach studies are network abstraction and network analysis (Derudder 2021). The heterogeneity in these studies mainly refers to the heterogeneous nodes (two types of nodes) or the statistical characteristics portrayed by the nodes in the network that are related to the network structure analyses like the node degrees, network connectedness and centralities.

When abstracting a real-world system into a bipartite network, the natural features of the nodes should be taken into consideration. These features could be the environmental variables, species specific variables, geographical variables of locations, epidemiological variables, biological variables, depending on the domain and objectives of the studies. These features of real-world phenomena contribute to the way interactions happen between the unlike nodes in a bipartite network, which eventually impact the network structure and its subsequent network statistics. Nevertheless, studies employing the bipartite network approach that incorporate the features of individual node are scarce.

O’Sullivan and Manson (2015) stipulate the studies surveying urban systems using network approach are ontologically and epistemologically unique from network studies conducted by physicists, and thus warrant a distinct methodology. Likewise, although the bipartite network approach studies between researchers that do not incorporate the natural features and those that do are methodologically identical to a certain degree, there are discernible ontological and epistemological differences between them. This has resulted in difficulties in employing the methodologies used for typical bipartite network studies. As a result, this study proposes a methodology framework for studies that intend to incorporate features of individual nodes to capture and model the interactions between the unlike nodes, employing a bipartite network approach. It is termed the bipartite network modeling (BNM) framework. Two case studies that fall under the disease transmission networks—mosquito-borne disease hotspot—and ecological networks—habitat suitability of a marine mammal—are presented to show the applicability of the framework.

The proposed framework could serve as an alternative to the typical system development life cycle (SDLC) for bipartite network modeling study that intends to incorporate the unique features of the distinct bipartite nodes. The contributions of this methodology framework include: a) specifying the need to check for the feasibility of employing a bipartite network approach, to determine the functional definitions of the nodes, links and the overall graph, to resolve the parameterization and the assumptions of the features of the bipartite nodes in the first stage; b) detailing the need to quantify the heterogeneous properties of the bipartite nodes; c) accentuating the necessity to scientifically evaluate many available quantification methods represented as the library of quantification techniques where computational intelligence approaches should be considered because the real data are always dynamic and complex; and d) specifying the network evaluation technique library to show that the verified and validated bipartite network model formulated can then be evaluated using both the typical and also novel network analysis where appropriate.

Therefore, the main contribution of this paper is the methodology framework serving as a generic methodology for researchers who intend to employ the bipartite network modeling approach across research areas and domains. It is for studies that aim to capture the heterogeneous nature of features in individual vertices that are contributing to the behavior of the network formed. The rest of this paper is organized as follows: “Bipartite Network Modeling (BNM) Framework” section introduces our proposed BNM methodology framework. “Dengue Hotspot Identification” and “Preferred Habitat of Irrawaddy dolphin (Orcaella brevirostris) at Kuching Bay” sections present the two case studies. In “Discussion” section, discussions are presented for the proposed BNM methodology framework with respect to the case studies presented with elaborations on the possible future works. Lastly, our conclusions are presented in the last section.

## Bipartite network modeling (BNM) framework

**Fig. 1:**
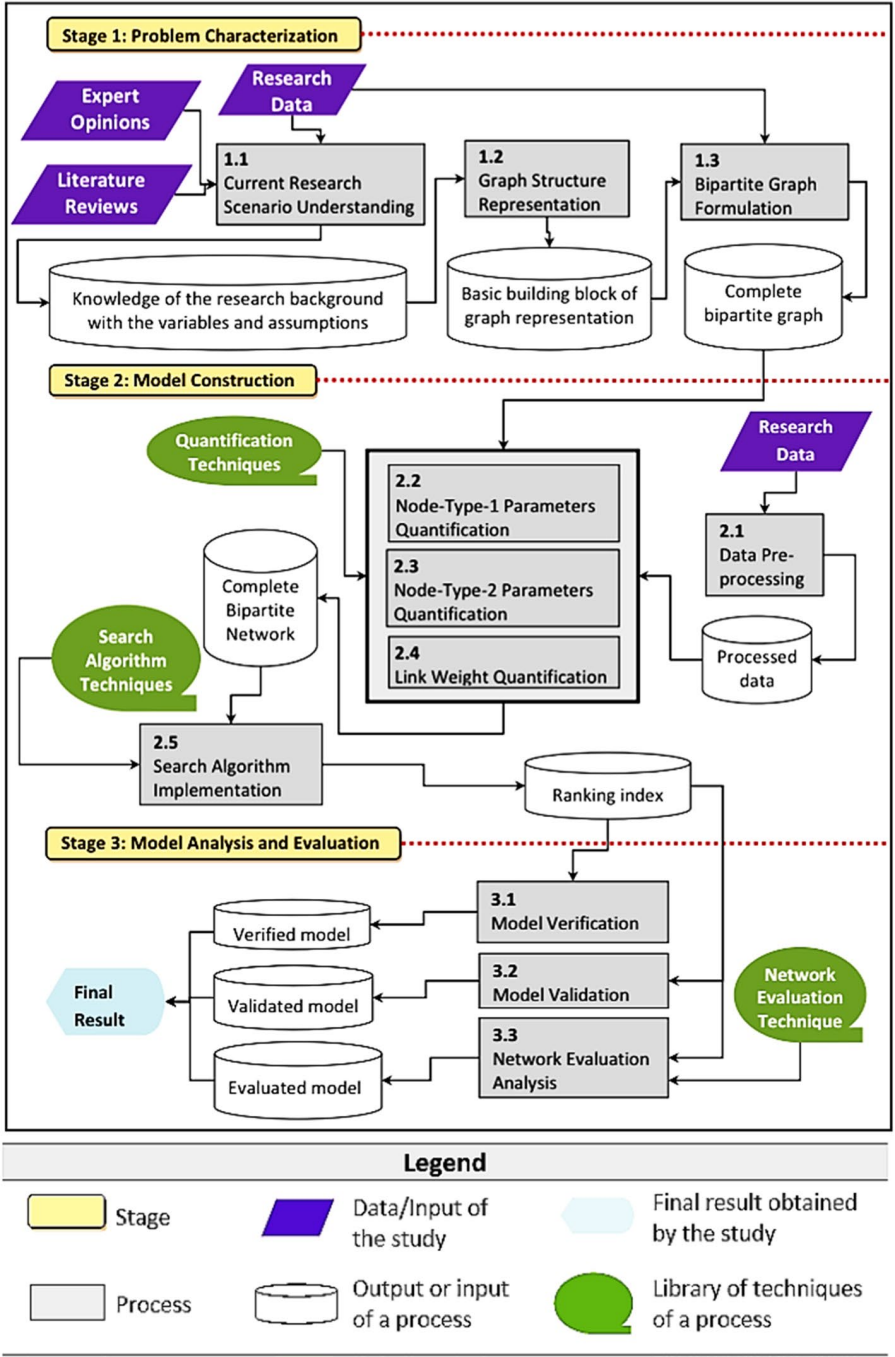
Bipartite Network Modeling (BNM) Framework

The BNM framework depicted in Fig. 1 captures the complete process of modeling. It has three distinct stages. The methodology is iterative and process-oriented in nature. The purpose is to formulate a validated bipartite network model that is able to rank either one or both the nodes, which is the hotspot (entity of interest according to the problem domain). Principal processes are detailed in every stage to guide the modeling activities. These processes are numbered in sequential order based on the stage they belong to. For example, process 2.4 refers to the fourth process of the second stage, the Link Weight Quantification. Every process produces one composite output. The output from one process serves as the input to the following process. The description of every stage and its corresponding processes are presented in the following subsections.

### Problem characterization

In this first stage are three main processes. They are current research scenario understanding, graph structure representation, and bipartite graph formulation. The focus of this stage is to formulate the graph structure for the network model of a research.

#### Current research scenario understanding

Denoted as process 1.1 in Fig. 1, the purpose of this process is to gauge the understanding of the current state of the research scenario. It is achieved through a triangulation procedure. They include discussions with the experts or stakeholders of the field, consolidating results from the review of the past literature, and studying research data. At the same time, the features or characteristics of the bipartite nodes that are significant in contributing to solving the research problem are identified. These features are the potential variables of the bipartite nodes of a study. The assumptions to be adopted in the study are also identified. The finalized variables and assumptions will be decided at the second stage.

#### Graph structure representation

**Fig. 2:**
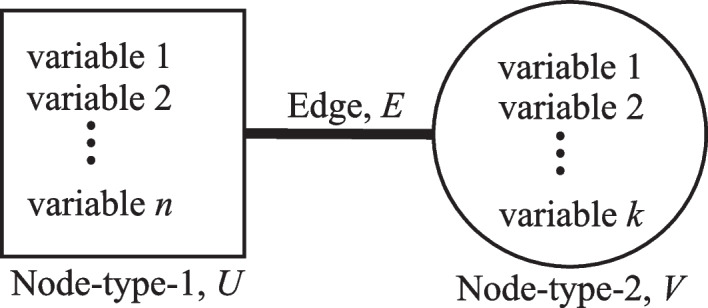
Representation of basic building block

The aim of the second process, process 1.2 in Fig. 1, is to identify the basis of the graph structure representation for the network system being studied. It is achieved by setting up the basic building block (Fig. 2), which is the simplest form of a bipartite graph that consists of two nodes (one from each bipartite node)—node-type-1 (*V*) and node-type-2 (*U*) as seen in Fig. 2—and an edge that joins them. Figure 2 shows that there are *n* and *k* features captured as variables for node-type-1 and node-type-2, respectively.

How the edge of a bipartite graph is defined depends on the research domain and the problem it is solving. For example, the edge could occur when there exist a virus-vector-host, supplier-manufacturer, manufacturer-contractor, cyberspace domain-host, twitter user-hashtag, non-volatile-and-volatile wine compound, location-pollutant, plantation-location, and species-habitat relationship. Next, the third process is important in formalizing and defining the bipartite nodes and the link through the information collected from the first process.

#### Bipartite graph formulation

For the third process, denoted as process 1.3 in Fig. 1, the research data obtained is used to form the bipartite graph using the basic building block formalized in the previous process. The complete bipartite graph representing the research scenario produced at this point is the output of the first stage. In addition, the potential variables of the study identified and the assumption to be adopted are also the pertinent output from the first stage. Mathematical and graphical representations of the research problem are thus formulated. The general mathematical expression of a formulated bipartite graph, *G*, having *i* number of node-type-1 denoted as *U* and *j* number of node-type-2 denoted as *V*, with *k* number of edges denoted as *E* connecting only node *U* and node *V*, is given in Eq. 1.1

### Model construction

The second stage comprises five processes: data pre-processing, node-type-1 parameters quantification, node-type-2 parameters quantification, link weight quantification, and search algorithm implementation.

**Data Pre-processing** The aim of the first process, denoted as process 2.1 in Fig. 1, is to ensure that the data is complete and balanced especially when real data is used. The output of this process is data that is ready to be used by the next processes.

#### Node-Type-1 parameters quantification and Node-type-2 parameters quantification

Both the second and third processes, denoted as processes 2.2 and 2.3 in Fig. 1, involve the parameters quantification of the nodes. Node-type-1 and node-type-2 refer to the respective bipartite nodes of the bipartite graph formulated in the first stage. The potential variables purportedly govern the behavior of a node within the network system have already been identified in the first stage. For instance, the *n* variables of node-type-1 and *k* variables of node-type-2 in Fig. 2. Hence, the focus of these two processes is to identify the techniques to quantify the parameters.

The mean to quantify a parameter depends on, amongst others, the research objectives, research domain or field, the past surveys, preference of researcher, and availability of traditional, novel, or emergent quantification techniques. One notable potential quantification method we wish to highlight here is the computational intelligence technique that is powerful and promising in tackling complex real-world problems. As there are a huge number of techniques to choose from, our methodology has resorted to represent this collection of choices through a library. It is termed as the quantification techniques library, denoted as a green-color shape in Fig. 1. From this library, the researcher shall consider quantification techniques deemed appropriate through scientifically sound procedures or analyses. Values for parameters of the bipartite nodes are then generated and computed using these techniques or taken from the research data.

#### Link weight quantification

The fourth process, denoted as process 2.4 in Fig. 1, intends to identify a quantification technique for determining the link weight. The link that connects the bipartite nodes has already been formalized and defined in stage one. As in their counterpart in the second and the third processes of the second stage, the researcher identifies these potential quantification techniques from the review of related work in their respective research domain. These potential techniques are also collectively represented as the quantification techniques library as shown in Fig. 1. As discussed in section two above, there is a lack of studies that compute the edge or link weight by incorporating the distinct parameters of each feature or variable characterizing both node types (captured by their respective variables). Consequently, the quantification of the link weight ought to consider this alongside capturing the complex interactions among nodes of both types which are given by the edge set (E) in Eq. 1.

The finalized quantification technique used in the study requires repetitive validation, and verification too if needed. Computation of the weight for each link is then executed. The BNM methodology framework presented in Fig. 1 shows that processes 2.2, 2.3 and 2.4 are grouped together, signifying that together they are responsible for producing the complete weighted bipartite network of a study.

#### Search algorithm implementation

The last process of the second stage uses a search algorithm to determine the ranking of one or both node-types. The nodes that are ranked at the top are the hotspots for the study. As revealed in Fig. 1, a green-colored shape named *search algorithm technique library* is connected to this process. This library symbolizes that there are many different search algorithms available in the research community for use. Among them are the two well-established and widely used web-based search algorithm: graph-theoretic based PageRank (Borgatti et al. 2002), Hypertext Induced Topic Selection (HITS) (Kleinberg 1999), and their variations that are extensively applied in influence calculation and network related research for nodes ranking purpose (Liao et al. 2020; London and Csendes 2013).

On the other hand, a study identifying malaria transmission hotspots reported that web-based HITS search algorithms (Eze et al. 2014) are useful and could be applied in other domains employing a bipartite network modeling approach. The researchers stipulated that a web-based HITS search algorithm exhibits the existence of structural similarity among the social network, web graph and the malaria network. Web-based HITS algorithm particularly stands out because the search of HITS involves both authority and hub nodes (Liao et al. 2020) which is equivalent to the bipartite nodes in the bipartite network. Adapting the HITS algorithm with the web graphs and the preference network resulted in a hybrid search engine represented in Algorithm 1 (Eze et al. 2014). Hub refers to either one of the bipartite nodes (Node-Type-1 or Node-Type-2) that is intended to determine its ranking.

**Fig. 3:**
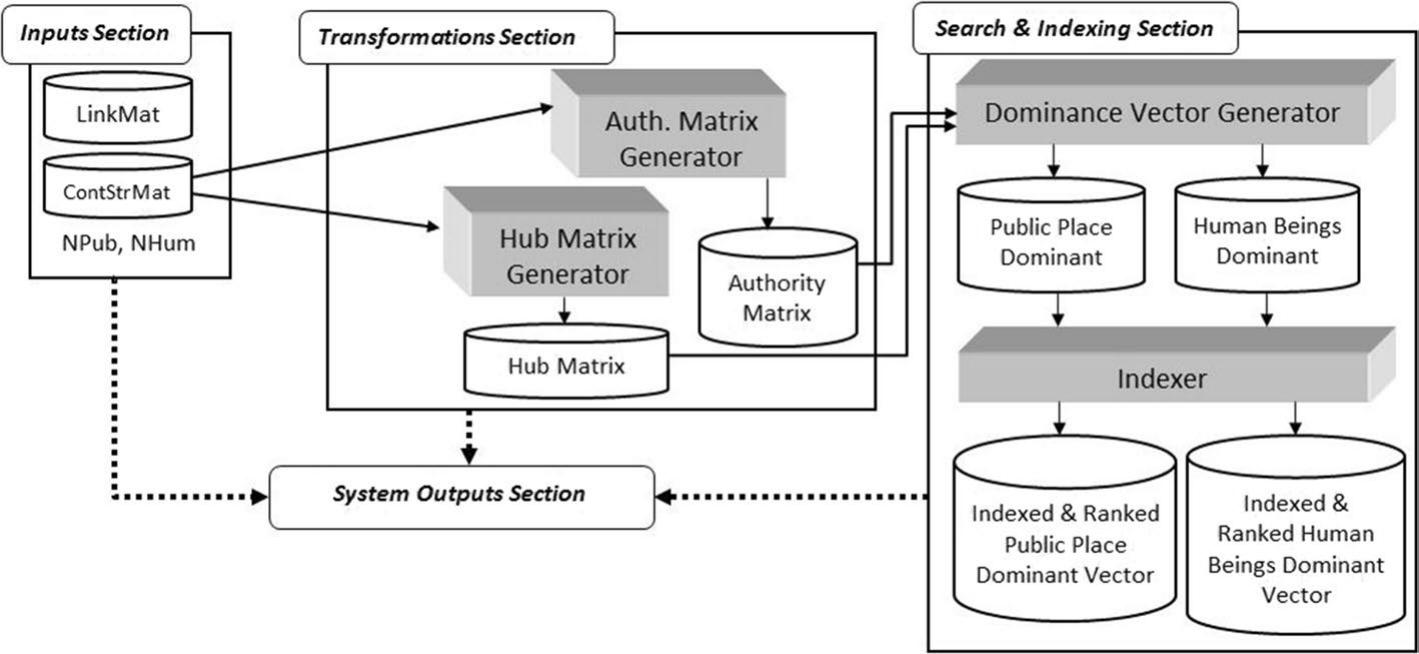
The search engine workflow (Source: Eze 2013)


The search engine model employed is made up of four main sections—the Input, Transformation, Search and Indexing, and the Output—as shown in Fig. 3. The hub and authority matrices refer to the bipartite nodes (Node-Type-1 and Node-Type-2) of the bipartite network system. The Input Section accepts the formulated bipartite network, in the form of two matrices—link matrix (LinkMat) and link weight matrix (ContStrMat)—and the number of nodes for each bipartite node in the Malaria Contact Network (Eze 2013)—public place node (NPub) and human being node (NHum) for the case of malaria network. The Transformation Section houses two generators—the Authority (Auth.) Matrix Generator and the Hub Matrix Generator. Both were used to generate the hub and the authority matrices respectively. The Search and Indexing Section is made up of the Dominance Vector Generator and the Indexer. The result of the operations in this section is the ranking of hubs which are the public places in terms of the malaria vector densities. The Output Section generates the result of the search engine operations, which are the hotspots of malaria transmission.

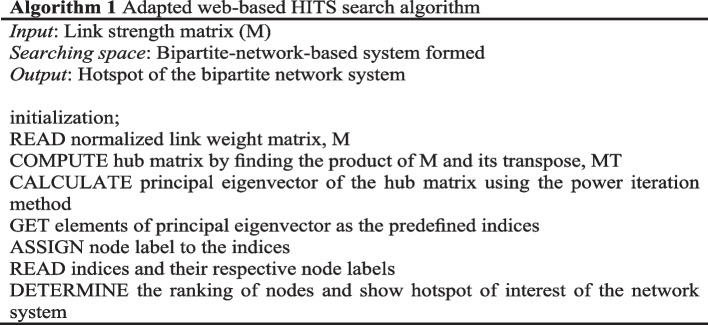


Consequently, the input for this process 2.5 is the complete bipartite network produced from the previous process 2.4. Implementing the selected search algorithm produces ranking indices where one or both bipartite nodes are ranked. The final result of stage two is the bipartite network model with the ranking of the nodes in the surveyed network system. Subsequently, the last stage, stage three, is elaborated next.

### Model analysis and evaluation

This last stage consists of three processes: model verification, model validation and network evaluation analysis. The main goal is to ascertain that the model formulated from the previous stage is verified, validated and evaluated.

#### Model verification and model validation

This process is denoted as process 3.1 in Fig. 1. The objective of model verification is to ensure that the research processes of modeling the network system in a study comply with the standard regulation, requirement or specification of a research community (IEEE 2011). To achieve this, the researcher generally uses other analysis systems and analytical methods as a benchmark to verify the implementation processes performed in the study. Conversely, model validation makes sure that the network system modeled meets the objective of the study and fulfills the needs of its stakeholders (IEEE 2011). Typical validation practices in modeling use real data or past survey results, or both to validate the result obtained in stage two.

Appropriate error analysis and comparative analysis are to be performed in these two processes to compare the actual model results or performance and the verification or validation results. Should the model fail to pass the verification or validation, or both processes, the model needs to be further refined by returning to the earlier stage(s) of the methodology. It marked the iterative nature of the BNM methodology.

#### Network evaluation analysis

Upon verifying and validating the bipartite network system modeled, it is passed to the third process of stage three, denoted as process 3.2 in Fig. 1. Network evaluation analysis aims to perform extended evaluations and analyses towards the model formulated. It further gauges the behavior, properties, structure, and function of the abstracted network system being studied. Typical complex network analysis methods, Petri nets methods for directed bipartite graph or network, existing and emergent analytical techniques, visualization tools that are scientifically sound are some examples for these purposes. In view of the numerous ways to analyze a network, they are collectively represented as a network evaluation techniques library in BNM methodology as shown in Fig. 1. The evaluation results obtained further validate and strengthen the research findings and provide auxiliary illustrations and insights for the research findings. The output of this stage, which is the final output of a study, is a verified, validated and evaluated bipartite network model.

The BNM methodology framework allows future expansion or extension of a formulated bipartite network model. This enables the researchers to extend their existing model when more data are acquired, implying that more nodes and edges are added to the network model. The framework also allows model expansion whereby additional variable(s) are required to be included. Likewise, the existing model can be modified when researchers intend to achieve another objective using the current model that they have. The researchers could refer to the BNM framework to identify the process (es) that need to be carried out when they want to perform any one of the above expansions. The BNM framework could act as a checklist as well so that proper modeling processes are carried out.

In the following sections, two studies employing the bipartite network approach following the BNM framework will be presented. The studies are in the fields of epidemiology and habitat suitability in ecology. The former study investigates the hotspot identification of vector-borne diseases whereas the latter detects the preferred habitat of a marine mammal species.

## Dengue hotspot identification

Hotspot detection of vector-borne diseases such as dengue is pivotal in ensuring the eradication (Aziz et al. 2014) of the disease concerned. Disease hotspots are geospatial areas with a high prevalence or efficient transmission of disease (Lessler et. al. 2017). Public health authority targets the hotspots to eliminate the vector effectively (Nagao et al. 2003; Ritchie and Johnson 2017). Dengue disease, like malaria, is one of the mosquito-borne diseases. The bipartite network approach is used to identify the dengue hotspots (Kok et al. 2018) where hotspots are defined as the public places of mosquito breeding sites. The BNM framework is adopted as the methodology in this study.

### Problem characterization of dengue hotspot identification

This section discusses the process in Stage 1 of the BNM framework (as seen in Fig. 1) which focuses on the formulation of the basic building block and then a graph structure representation of the intended bipartite dengue contact network.

**Fig. 4:**
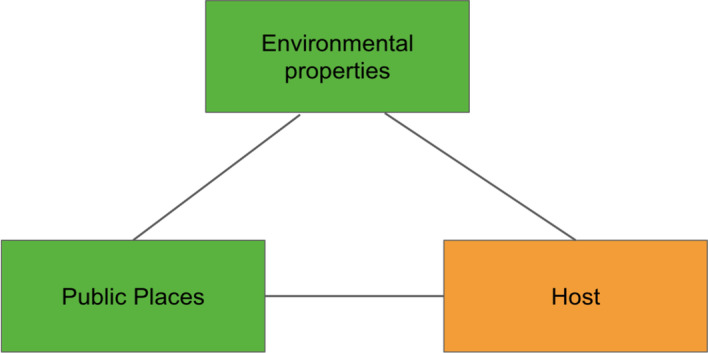
Epidemiological triangle (ET)

As demonstrated by Eze (2013), epidemiological studies that relate the interaction among environmental properties, public places and hosts can be visually represented as a graph consisting of three vertices and referred to as the epidemiological triangle (ET). Similarly, as depicted in Fig. 4, Kok et al. (2018 p. 3) use it as the basis for formulating the study on dengue transmission. The three epidemiologic factors in the epidemiological triangle are interdependent and used to identify the basic building block.

**Fig. 5:**
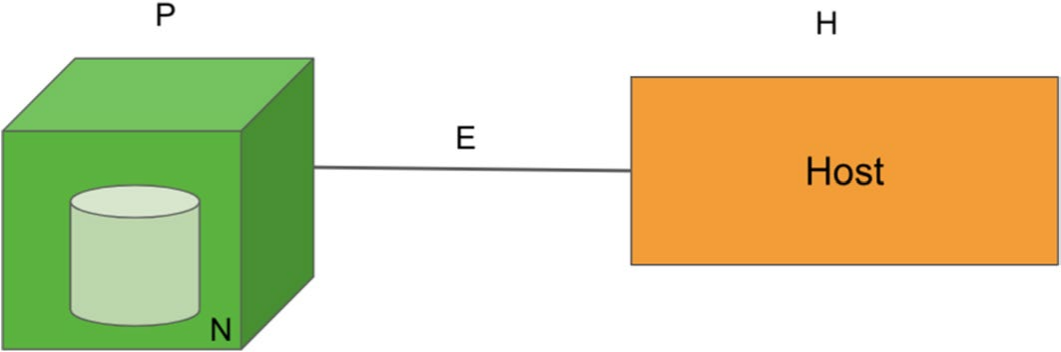
Basic building block of the bipartite dengue contact network

As a mosquito-borne disease, the main hosts in this study are mosquitoes and humans. However, the mosquitoes are not characterized as a node in the network model of this study as the mosquitoes are reported to be unable to fly further than 400 m from a particular public place (Eze et al. 2011). Therefore, it is assumed that the public place node houses the mosquito nodes resulting in the consideration of simply the public place. Besides that, public place and environmental properties are two risk factors that are strongly related where the public place component specifies the spatial features of specific environmental properties (Liew 2016). At the same time, environmental properties characterize a specific public place and differentiate a public place from another. Therefore, the public place component can be viewed as a component housing the environmental properties. Hence the ET is further modified, as shown in Fig. 5. Public place is denoted as P, a component of environmental properties denoted as N and the component of host denoted as H. The previous three vertex graph structure (Fig. 4) of ET is modified to a two-vertex graph structure as depicted in Fig. 5 (Kok et al. 2018 p. 3).

Based on the assumption that the public place component is strongly related to the environmental properties, forming the basic building block of the bipartite network model, as depicted in Fig. 5. The two different nodes consist of human and public place nodes. The component of the host is replaced with the human and denoted as H. There are two vertices, P and H, that imply the attributes for vertex public place (P) and vertex human (H) are different, signifying a bipartite network which is a heterogeneous network of two node types. Consequently, Fig. 5 shows a network structure that consists of the sets of public place nodes (P), human nodes (H) and edges (E). The set of edges is the link between the public place and human nodes. The graph structure is termed Bipartite Dengue Contact (BDC) graph, while the network model is coined as the Bipartite Dengue Contact (BDC) network.

Process 1.2 of the BNM framework ensures that a graph is different from a network where a BDC graph is an unweighted bipartite graph for a visual representation of the BDC network. BDC network is a weighted bipartite graph that gives the topological and functional relationship of the bipartite nodes and their respective links (Rayfield et al. 2011). The link weight is a measure of affinity between nodes in the BDC network. To quantify the contact strength values, potential parameters for public places and human nodes are identified as both the nodes are associated with the increased probability of dengue spread.

Two parameters are potential to be considered for the human nodes, namely the frequency of a patient visiting a public place (*Fh*), and the total duration of stay in a public place (*Du* in second, s). The parameters that are attached to public place nodes involve the mosquito characteristic, for instance, life cycle index (*Lc*), survival rate (*S*), and biting rate (*B*); environmental parameters, which includes, total precipitation amount (*Pre* in meter, m), humidity (*K* in percentage, %); geographical parameters, for instance, altitude (*Al* in meter, m); and frequency of a public place visited by humans (*Fl*). Based on the research data, the link between the nodes formed when humans visited public places.

For process 1.3 on Bipartite Graph Formulation in the BNM framework (Fig. 1), the complete BDC graph is formed in this process using the basic building block shown in Fig. 5. From process 1.1, a total of twelve Epidemiological Week (Epi Week 28 to 39) of dengue patients’ mobility data are collected. However, only data collected from the first two weeks of Epi Week (Epi Week 28 and 29) is chosen for this paper to demonstrate the formulation process of the BDC network model.

There are eight unique individual dengue patients and each of them is given a unique code. These eight unique coded individual dengue patients are consequently identified as the eight human nodes of the BDC network. They are labeled as H1, H2, H3, H4, H5, H6, H7, H8 where H is the symbol used for human nodes and the numerical number 1 and 8 is used to differentiate one human from another. The number of human nodes includes dengue positive and possible positive patients. Based on process 1.1, individuals will be registered when the patient visits the hospital or clinic due to fever, and Immunoglobulin M (IgM) dengue serology test will be conducted to diagnose dengue fever. The possible positive patient in this study represents the patient who has negative IgM dengue serology test results (obtained from the investigation form). It is revealed from the report by the Centers for Disease Control and Prevention (CDC) (2014) that the primary infection shows a slow and low titer antibody response compared to the secondary infection. Dengue IgM serology has low sensitivity during the early phase of dengue fever as the virus and IgM antibodies may be at undetectable levels for those who submit a day five acute specimen (CDC 2014). Therefore, the human mobility of the patient who has a negative IgM serology needs to be considered in this model.

As for the public place nodes, they were visited by both human nodes with dengue positive and possibly positive capability to provide a possible new risk public place to the model. Based on the eight human nodes which have been identified, there are a total of 19 public places visited by the eight human hosts. Thus, these public place nodes are labeled as P1, P2, P3, P4, P5, P6, P7, P8, P9, P10, P11, P12, P13, P14, P15, P16, P17, P18 and P19, where P is the symbol used for the public place node and the numerical number 1 until 19 is used to differentiate a public place from another. Each of the public place nodes is labeled with its corresponding latitude and longitude values.

**Fig. 6:**
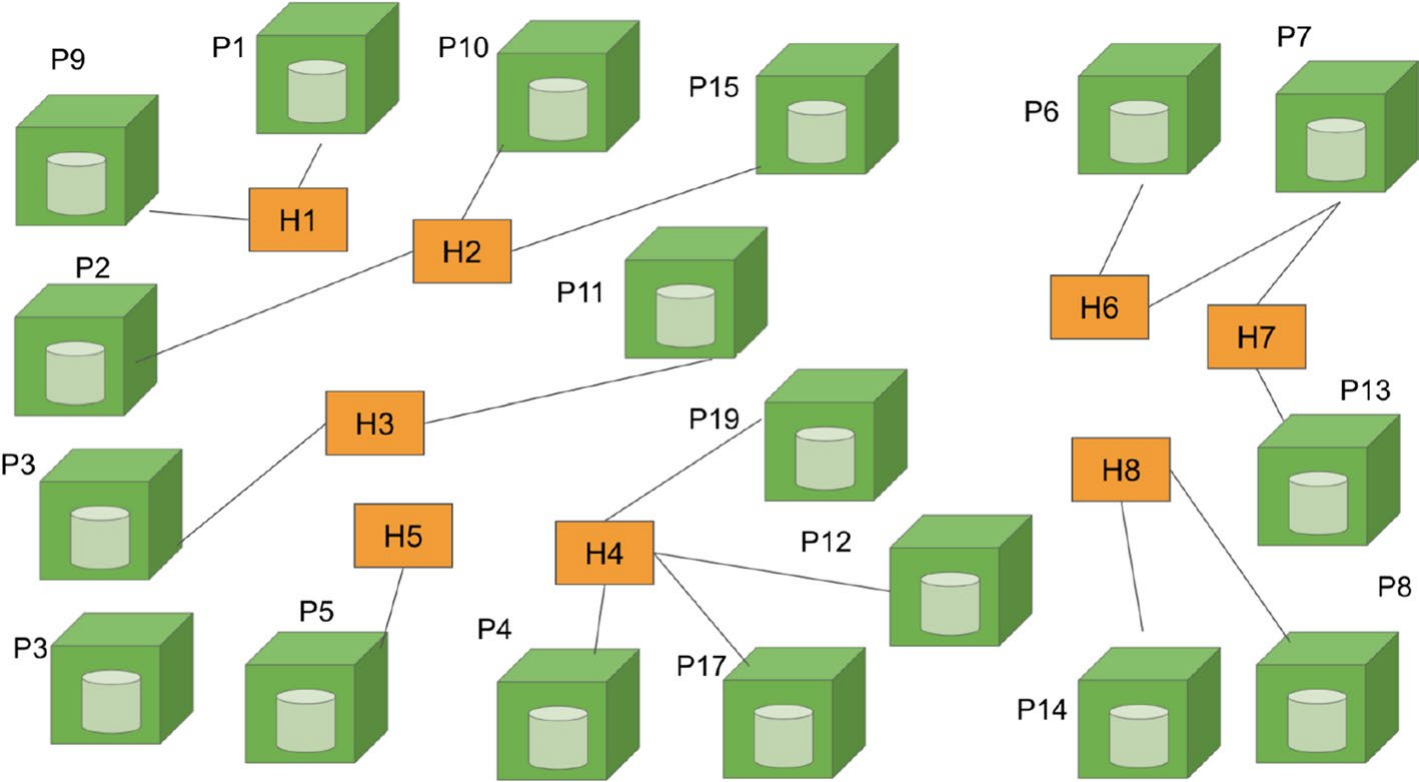
Complete bipartite dengue contact graph for dengue patients

Next is the identification of the link which joins the human and public place nodes. The link is formed when H visits P. To trace all links, each unique human host's movement visiting each public place is extracted from the investigation form. The information of links formed concerning each public place and each human can be identified and is revealed in the complete BDC graph for the BDC network presented in Fig. 6. BDC graph is the bipartite graph, denoted as BDC_DEN_KCH_ where set H, the human nodes, consists of eight elements, and set P, the set of public place nodes, consists of nineteen elements. Set E, the set of links that join elements of H and P, has 20 elements. This is given in Eq. 2 (Kok et al. 2018 p. 5). Subsequently, the BDC graph in Fig. 6 is a graph of 8H by 19P with 20 edges. The degree of each public place node is {1, 1, 1, 1, 1, 1, 2, 1, 1, 1, 1, 1, 1, 1, 1, 1, 1, 1, 1}. On the other hand, the degree of each human node is {2, 3, 3, 4, 1, 3, 2, 2}. The sum of the degrees of the public place or human nodes is 20, which is also the total number of edges in the BDC graph.2

### Bipartite dengue contact network model construction

This section discusses the processes in Stage 2 of the BNM framework (as seen in Fig. 1) which focuses on the formulation of the BDC network model. It explains the quantification of the parameters for the public place and human nodes, and the quantification of links connecting these two types of nodes.

The first process (denoted as process 2.1 in the BNM framework) at this stage is to process the data collected from the previous stage. The data obtained is raw and untidy, which requires data pre-processing for the public place and human nodes. This is an essential process to determine the parameters’ values attached to or defined for the public place and human nodes in the following processes.

### Public place node

The first step is to change the public place name into Global Positioning System (GPS) coordinates using Google Maps. An algorithm to call a user-defined function is used to calculate the distance of the new incoming public place and the existing public places from the data frame. A new public place node is declared in the database only if the distance between the new public place node and the current public place (in the database) is greater than 400 m because the maximum flight range of the *Aedes* mosquito is 400 m. Next, all the public places' GPS coordinates of the data frame are passed to a user-defined distance matrix generator where a matrix of geographical distances between public places is generated.

### Human node

The human node refers to the patient's identity and this can be obtained from the investigation form. To protect the patient's confidentiality, patient identity is replaced by an algorithm-generated ID. The BDC network model consists of eight human nodes. Thus, the human nodes are identified as H1, H2, H3, H4, H5, H6, H7, and H8.

### Parameters

Parameter values such as temperature, humidity, precipitation, and altitude are collected for node quantification. Pre-processing these parameter values is necessary to prepare the parameter in developing a robust model to quantify the nodes in the following processes.

The human mobility data capture the patients' movement two weeks before the onset date. In order to observe the effect of the parameters on the environment, the average temperature two weeks ago at that particular public place needs to be calculated. For instance, the onset date for the first patient (denoted as H1) was 2015–07-09. The human mobility data captured the movement two weeks ago, which is 2015–06-25 until 2015–07-08. The H1 visited P1 on 2015–06-25. Thus, the average temperature 14 days ago, between 2015–06-11and 2015–06-24, is calculated with the Eq. 3. In Eq. 3, the *i* represents the day before the human mobility date starts and *i* is a positive integer. Thus, *i* = 14 represents 14 days calculated from the human mobility start date and gradually decreases to *i* = 1, which is one day before the human mobility date. The variable *k* in Eq. 3 represents temperature, humidity, precipitation, or altitude.

3 $$Average_{k} = Average\left( {\mathop \sum \limits_{i = 14}^{1} k_{i} } \right)$$

With the pre-processed data, process 2.2 can be activated with the quantification of the parameter values of node-type-1, the public place nodes, specifically on the parameters namely the life cycle parameters, survival rate, biting rate and the frequency of humans visiting a public place. It is established that the mosquito vectors hardly move far away from their breeding sites. Thus, these vector activities taking place in that particular locality will affect the dengue transmission. The activities below have been considered in this study to model dengue transmission.

i. Number of days to complete a mosquito vector life cycle that will affect the dengue transmission rate. Thus, the vector life cycle duration model needs to be constructed.
ii. Mosquito survival rate is also affecting dengue transmission. As the higher the survival rate resulted in higher mosquito population, and hence the higher transmission rate. Thus, the vector survival rate model is incorporated into the contact network.
iii. Mosquito biting rate affects dengue transmission, as the more frequent the mosquito bites the human host, the higher the probability the dengue spreads. Thus, the vector biting rate model is constructed.

#### Quantification for duration of vector life cycle

The duration of the vector life cycle measures the life cycle duration of mosquito from an egg to an adult at every locality in a BDC network. Due to its known dependence on temperature, the life cycle of the *Aedes* mosquito plays an essential role in understanding the effects of environmental property on dengue transmission (Carrington et al. 2013). The life cycle duration is negatively associated with the temperature. Vector life cycle duration parameter is thus termed the vector life cycle index with symbol *Lc*. This vector life cycle index, valued between 0 and 1, is defined as the measurement of the life cycle duration of the *Aedes* mosquito. Development of *Lc* is presented in Kok et al. (2018). It is a temperature-dependent model formulated as a function of *t* where *t* represents temperature, with a degree of 6 as in Eq. 4 (Kok et al. 2018, p. 5).

4 $$Lc = - 0.633t^{6} - 0.786t^{5} + 1.488t^{4} + 1.153t^{3} - 0.408t^{2} - 0.758t - 0.504$$

After the values of *Lc* for each public place node are computed, the inverse of it, 1/*Lc* is to be used in the link weight quantification later. This is because the life cycle index is inversely proportional to the dengue transmission rate, where the shorter the time taken for a complete life cycle leads to an increase in the dengue infection rate.

#### Vector survival

The survival parameter measures the survival probability at a locality to indicate the vector survival rate at one locality. This parameter is included to account for the importance of dengue transmission as one of the significant contributors to the vector hotspot (Lambrechts et al. 2011). The mosquito survival rate is positively associated with temperature (Rueda et al. 1990; Tsai et al. 2017; Lee and Farlow 2019). Nevertheless, there is no documented source for vector survival data. Quantification of the vector survival parameter is given in Kok et al. (2018). Vector survival parameter is termed the vector survival index with symbol *S*. In this study, generating a vector survival index is the same as the process to generate the life cycle index. The resulting model is given in Eq. (5) (Kok et al. 2018, p.6). It is then used to compute the value of *S* for each public place node.

5 $$S\left( t \right) = 1.3908t^{6}- 0.2951t^{5}- 3.8642t^{4} + 1.3217t^{3} + 1.2971t^{2}- 0.1412t + 0.591$$

#### Vector biting

One of the crucial activities, like biting, contributes to dengue transmission (Phaijoo and Gurung 2015; Wesolowski et al. 2015). Scott et al. (2000) associated the temperature and blood-feeding frequency of female *Ae. Aegypti*. This blood-feeding frequency indicates the number of blood meals the mosquito takes, referring to the number of mosquito bites. A linear regression model of the blood-feeding is derived and given in Eq. 6 that represents the total mosquito biting rate per week where *T* represents the average weekly temperature range from 21℃ to 32℃ in the study area, Thailand.

6 $$B(T ) = 0.03T + 0.66$$

As the unit of biting rate in this study is the daily mosquito biting rate, Eq. 6 is divided by 7 to transform it into a daily biting rate. The modified biting parameter model is given in Eq. 7. Scott et al. (2000) applied the model when the temperature ranged from 21 to 34. If the temperature is out of this range, the biting parameter is a baseline value, 0.8 (Scott et al. 2000). Subsequently, the vector biting index, *B* of this study is computed using the average temperature of the particular date and public place.

7 $$B\left( T \right) = \left\{ {\begin{array}{*{20}l} {0.004286T + 0.09429, } \hfill & {21^{ \circ } {\text{C}} \le T \le 32^{ \circ } {\text{C}}} \hfill \\ {0.8,} \hfill & {{\text{otherwise}}} \hfill \\ \end{array} } \right.$$

#### Frequency of public place visited

This study includes the number of times the dengue patients visit a public place as one of the parameters for public place nodes in the BDC network to capture the effect of visiting the dengue patients in a public place. This parameter is termed the frequency or number of times one human visits a public place and is denoted as *Fl*. A link matrix, Link_Mattrix_BDC Network_ is created to record the *Fl* and is defined in Eq. 8. It is used to generate the link matrix for this study. Another parameter, *Fh* measures the number of times that a human visited a public place and is discussed in the next section. Therefore, *Fl* is defined as in Eq. 9.8

9 $$\begin{gathered} {\text{Fl}}_{i} = \mathop \sum \limits_{j = 1}^{8} \left[ {{\text{Link\_Matrix}}_{{{\text{BDC}}\;{\text{network}}}} \left( {{\text{P}}_{i} {\text{H}}_{j} } \right) \times {\text{Fh}}_{ji} } \right] \hfill \\ {\text{where}}\;i \in \left\{ {1, 2, \ldots , 19} \right\}j \in \left\{ {1, 2, \ldots , 8} \right\} \hfill \\ \end{gathered}$$

Four significant public place parameters namely *Lc*, *S*, *B* and *Fl*_*i*_ are explained. *Lc* and *S* are quantified through polynomial curve fitting with three attributes: latitude (*x*), longitude (*y*) and temperature (*T*). *B* is quantified using a linear step function with respect to temperature (*T*) and *Fl*_*i*_ is quantified through the Eq. 9 defined earlier. Parameters initially decided for the public place node of the BDC network are then further refined. It is crucial to keep the model simple (Barnes and Fulford 2014 p. 3). Thus, latitude, longitude and temperature are excluded from the public place parameter as their effects have been accounted for in the life cycle model, survival model and biting rate.

The output of process 2.2 (Fig. 1) is the seven parameters finalized as the BDC network public place node parameters. They are life cycle index, survival index, biting index, humidity, precipitation, altitude, and number of times a public place is visited by humans. The values of three parameters—*Al*, *Pre* and *K*—are directly obtained from the research data. Table 1 presents the values of all seven parameters for each of the 19 public place nodes in the BDC network dataset.

**Table 1:** Values of the seven parameters of BDC Network

Public Place Node	*Lc*	*S*	*B*	*AI*(m)	*K* (%)	Pre (mm)	*FI*
P1	0.1995	65.1060	0.8	13	76.3	2.2	1
P2	0.1817	78.1297	0.9	24	74.2	0.1	1
P3	0.2000	68.8743	0.8	7	85.3	13.1	1
P4	0.2008	64.4377	0.8	8	79.3	7.7	1
P5	0.1791	80.9369	0.8	20	78	1.1	1
P6	0.1791	80.9369	0.8	4	78	1.1	1
P7	0.2024	63.9387	0.8	9	78.8	5.4	2
P8	0.1709	91.3251	0.8	6	76.1	0.7	1
P9	0.1995	65.1060	0.8	13	76.3	2.2	1
P10	0.1817	78.1297	0.8	54	74.2	0.1	1
P11	0.2043	66.5415	0.8	9	82.6	11.6	1
P12	0.1977	65.9558	0.8	4	77.7	5.3	1
P13	0.1994	65.1060	0.8	21	76.3	2.2	1
P14	0.2010	64.4377	0.8	8	77.4	5.3	1
P15	0.1817	78.1297	0.8	28	74.2	0.1	1
P16	0.2043	66.5415	0.8	8	82.2	12.3	1
P17	0.2010	64.4377	0.8	10	76.9	5.3	1
P18	0.1791	80.9369	0.8	25	75.3	0.6	1
P19	0.1977	65.9558	0.8	9	76.1	0.1	1

Next, process 2.3 begins where two parameters are identified for the human node of a BDC network, namely, time duration of human stay at a public place, *Du*, and the frequency of a human visiting a public place, *Fh*.

#### Time duration of stay of human at a public place

The total duration of a human stay at a public place across 14 days is recorded in the investigation form. These 14 days are the periods of dengue patients before the first symptoms and the dengue patients' movement within these 14 days is essential in dengue transmission (World Health Organization (WHO) 2012). The duration is recorded in either day, hours or even minutes. The time taken of a human stay at a public place across 14 days is calculated in seconds. Denoted as *Du*_*ij*_, the duration for human *j* visited a public place *i* across 14 days is calculated using Eq. 10 and the values are given in Table 2.

10 $${\text{Du}}_{{{\text{ij}}}} = {\text{total}}\;{\text{duration}}\;{\text{of}}\;{\text{human}}\;j\;{\text{visited}}\;{\text{public}}\;{\text{place}}\;i\;{\text{in}}\;{\text{seconds}}$$

**Table 2:** Values of parameters for human nodes for the BDC Network

P node	H node	Du_*ij*_ (s)	P node	H node	Du_*ij*_ (s)	P node	H node	Du_*ij*_ (s)
P1	H1	655,200	P7	H6	37,800	P12	H4	207,000
P2	H2	705,600	P2	H7	997,200	P13	H7	126,000
P3	H3	990,000	P8	H8	918,000	P14	H8	291,600
P4	H4	284,400	P9	H1	529,200	P15	H2	75,600
P5	H5	756,000	P10	H2	428,400	P16	H3	57,600
P6	H6	1,146,600	P11	H3	162,000	P17	H4	14,400

#### Frequency of human visiting a public place

The parameter of the number of times a human visited a public place is represented by the symbol *Fh*. Human and public places here refer to each of the respective public places and human nodes. This parameter is denoted as *Fh*_*ij*_ and is defined in Eq. 11 to record how many times has human node *j* visited public place node *i*.

11 $$Fh_{ij} = \left\{ {\begin{array}{*{20}l} n \hfill & {{\text{if}}\;{\text{H}}_{j} \;{\text{visited}}\;P_{i} \;n\;{\text{times}}\;{\text{where}}\;n \in Z^{ + } } \hfill \\ 0 \hfill & { {\text{if}}\;{\text{H}}_{j} \;{\text{did}}\;{\text{not}}\;{\text{visit}}\;P_{i} } \hfill \\ \end{array} } \right.$$

The value of *n* and thus *Fh*_*ij*_ for every human node to each of the 19 public place nodes is determined by tracking the movement of each unique individual human. The values of *Fh*_*ij*_ agree with the number of link(s) formed between human node *j* and public place node *i* in the BDC graph shown in Fig. 6. The values of *Fh*_*ij*_ are provided in Table 3.

**Table 3:** Values of *Fh*_*ij*_ for human nodes for BDC Network

*Fh*		*j* (Human Node)							
		1	2	3	4	5	6	7	8
$i$(Public places)	1	1	0	0	0	0	0	0	0
	2	0	1	0	0	0	0	0	0
	3	0	0	1	0	0	0	0	0
	4	0	0	0	1	0	0	0	0
	5	0	0	0	0	1	0	0	0
	6	0	0	0	0	0	1	0	0
	7	0	0	0	0	0	1	1	0
	8	0	0	0	0	0	0	0	1
	9	1	0	0	0	0	0	0	0
	10	0	1	0	0	0	0	0	0
	11	0	0	1	0	0	0	0	0
	12	0	0	0	1	0	0	0	0
	13	0	0	0	0	0	0	1	0
	14	0	0	0	0	0	0	0	1
	15	0	1	0	0	0	0	0	0
	16	0	0	1	0	0	0	0	0
	17	0	0	0	1	0	0	0	0
	18	0	0	0	0	0	1	0	0
	19	0	0	0	1	0	0	0	0

Seven parameters are identified and quantified for the public place node and two parameters are determined for the human node. Processes 2.2 and 2.3 in Stage 2 of the BNM framework are completed.

Once both distinct node types have been quantified, the link between them can now be quantified and is depicted as process 2.4 in the BNM framework. Twenty link edges are identified from the BDC graph established earlier and the weights of these edges need to be computed. This link weight is termed dengue contact strength (DCS), representing the link affinity between the human and public place nodes. The stronger the strength indicates the greater degree of attachment between the human and the specific public place, which contributes to a higher degree of human contact with the specific public place.

Eze (2013) introduced a summation rule to compute the contact strength for the edge formed between the bipartite nodes of the malaria transmission network. The quantification technique is explained in Eq. 12. Summation is used because the total of the individual parameters will contribute to a more significant value, indicating a stronger strength. Since individual parameters consist of rational numbers such as 0.7, the product of these rational numbers will contribute a smaller value which indicates a weaker strength. Thus, a summation rule is the most suitable one. DCS_*ij*_ refers to the dengue contact strength of the link formed between public place *i* and human *j*.

12 $$\begin{aligned} {\text{DCS}}_{ij} & = \left( {\sum {{\text{PublicPlace}}\_{\text{Node}}\_{\text{Parameters}}_{i} } } \right) + \left( {\sum {{\text{Human}}\_{\text{Node}}\_{\text{Parameters}}_{ij} } } \right) \\ & = (Lc_{i} + S_{i} + B_{i} + Al_{i} + K_{i} + \Pr e_{i} + Fl_{i} ) + \left( {Du_{ij} + Fh_{ij} } \right) \\ \end{aligned}$$

**Fig. 7:**
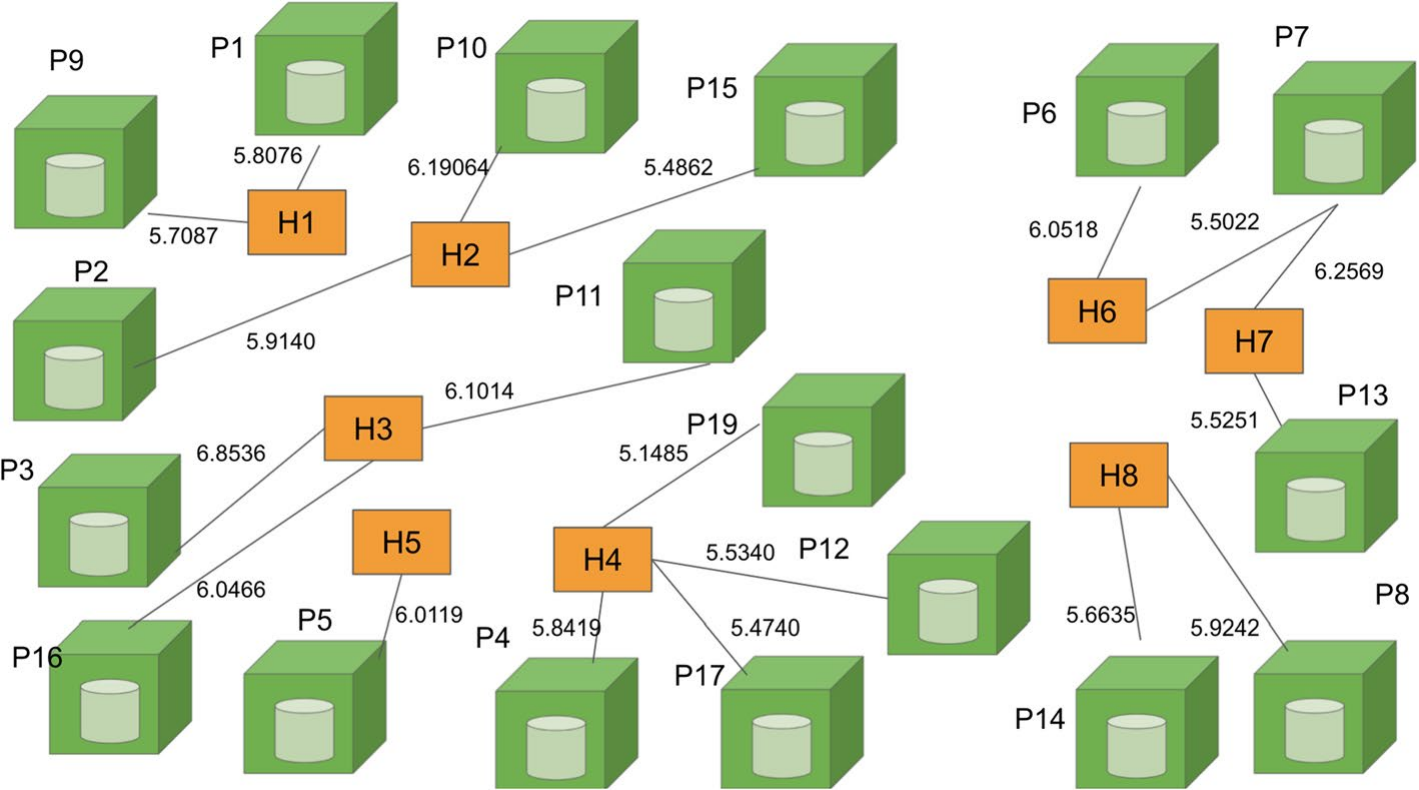
Bipartite Dengue Contact (BDC) Network

Using the normalized parameter values of *Lc*, *S*, *B*, *Al*, *K*, *Pre* and *Fl*, the complete DCS is computed. The output of DCS for all links in the BDC graph (Fig. 6) eventually resulted in the BDC network as shown in Fig. 7.

The BDC network represented in the form of a matrix (where the row of this matrix is the location node while the column represents the human node, and the elements of the matrix are the normalized link weight) is the input of process 2.5 in the BNM framework corresponding to the implementation of the search algorithm. Similar to the previous studies that adopt the framework, this study also used the HITS search algorithm that involves the computation of principal eigenvalues and eigenvectors. The implementation of the algorithm involves fours steps namely, the generation of hub and authority matrices; the generation of the corresponding principal eigenvectors; the assignment of nodes’ labels according to the principal eigenvectors and the assignment of the dengue hotspot ranking (DHR) values; and finally the generation of the output of the algorithm which is the locations prioritized according to the DHR values (Fig. 3). The higher-ranking location represents the more critical the location is in terms of dengue control intervention.

The final stage of the BNM framework is the Model Analysis and Evaluation of the Bipartite Dengue Model. The model is verified via a comparison of the Root-Mean-Square Error (RMSE) made with a benchmark system, that is the UCINET 6 for Windows, a powerful network analysis software (Borgatti et al. 2002). The analytical verification is conducted by calculating the Spearman’s Rank Correlation Coefficient (SRCC) between the hub matrix and the DHR values. The validation process was executed by calculating the SRCC between the targeted and validated network. Further analyses like the predictive power analysis, parameter significance analysis and data size analysis were also conducted as reported in Kok et al. (2018, 2019).

## Preferred Habitat of Irrawaddy dolphin (*Orcaella brevirostris*) at Kuching Bay

Irrawaddy dolphin (ID) (*Orcaella brevirostris*) is listed under the category and criteria of Endangered A2cd + 3cd + 4cd (version 3.1) where it has been categorized as Vulnerable A4cd (version 3.1) since 2008 by the International Union of Conservation of Nature and Natural Resources (IUCN) Red List of threatened species (version 2017) (Minton et al. 2017). However, the sub-population of ID at Kuching Bay, Sarawak, Malaysia is not listed in the databases of the IUCN until the year 2017. No established and consistent scientific survey and research has been conducted on the distribution and abundance of the ID at Kuching Bay (Peter 2012) until the commencement of the Sarawak Dolphin Project (SDP) in 2008 by the Institute of Biodiversity and Environmental Conservation (IBEC) of Universiti Malaysia Sarawak (UNIMAS).

The habitat suitability related studies reviewed (Clauzel et al. 2018; Heinonen 2019; Torres et al. 2017) always relate the abundances of a species with the environmental properties. The approaches employed are predominantly statistical, which demand a big data size whereas the deterministic approaches used by population dynamics studies incorporate the aspect of habitat suitability into their modeling effort (Cayuela et al. 2020; Marquez et al. 2021; Nusz et al. 2018). The deterministic approaches are based strongly on established physical or mechanistic laws and require detailed species-specific demographic values. Apart from this, generalization is mostly assumed and incorporation of features from individual habitat location or species, or both could hardly be found in these approaches.

The above approaches are not suitable to be applied in this study as the data that the study had is scarce. The reason is the lacking scientific and detailed demographic information about ID at Kuching Bay and suitable physical law to be applied in modeling habitat suitability of a species at the time the study is carried out. Furthermore, this study intends to incorporate the attributes of individual habitat location or species, or both. Nevertheless, the graph-theoretic network modeling approaches is not restricted by these limitations.

### Problem characterization of preferred habitat

In this section, discussion, and justification for the formulation of basic building block as the graph structure representation for the intended bipartite habitat network are presented.

**Fig. 8:**
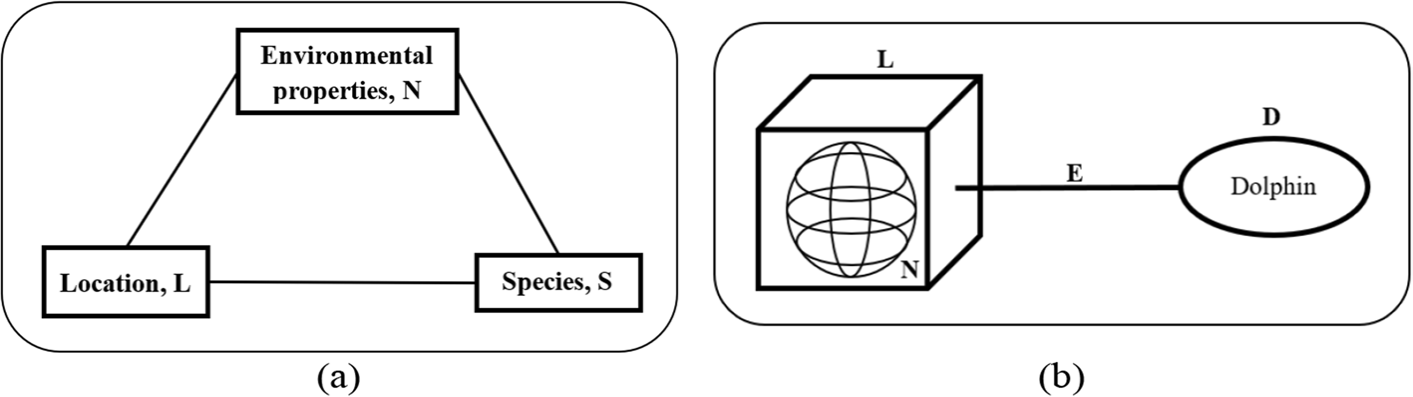
**a** Habitat Suitability Triangle (HST) (Source: Liew et al. 2015a, p. 268); **b** Basic Building Block of the Bipartite Network (Source: Liew et al. 2015a, p. 268)

Habitat suitability studies reveal that species, location, and environmental properties are the three typical main components in its research structure. These graph-structure-like components show that the location and environmental properties components, and the species’ component are of two different natures sharing different attributes. It is termed the Habitat Suitability Triangle (HST) in this study (Fig. 8a). The heterogeneous nature suggests the bipartite network approach could be applied and the BNM framework presented in Fig. 1 could be used to guide the modeling processes. The data this study has are real-world data collected by the SDP team (Peter 2012), which consist of four main sub-datasets. The data record individual ID identified by SDP; the ID re-sight’s maps (Peter 2012, Fig. 4.3c and 4.3d, p. 68); the physical and water parameter readings and sighting of ID at each data collection point; and species sighting data at the location point whenever ID are sighted. To triangulate these data that are scarce, imbalanced, and without scientific information, opinions from the experts in the field of animal nutritional ecology, and the researchers of the SDP team are also collected. Three main assumptions adopted in this study include every individual ID at Kuching Bay is free to settle anywhere, and the territoriality and preemption by early settlers do influence settlement of other individual ID (Fletcher Jr. et al. 2011), is physically fit and possess the ideal capability to assess the quality of all locations available and locate their most preferred habitat (Fletcher Jr. et al. 2011), and possess prior knowledge which optimizes the foraging behaviors of each ID with the least trade-off of meeting predator.

Since the environmental properties (*N*) explain the physical characteristics of a location (*L*) and are thus inseparable, HST is further modified into a two-node graph as depicted in Fig. 8b. It is used to form the basic building block of the network structure of this study. The basic building block consists of two nodes: the dolphin node (D) representing the ID species under study and the location node (L) representing the location with its unique environmental properties (N) enclosed within; and a link (E) that joins the two nodes. The link is formed when a D visits an L. The link weight is termed the Habitat Suitability Strength (HSS). It represents the relationship between the location and dolphin nodes where greater link strength represents stronger affinity between the dolphin and the specific location, which implies higher suitability of this location to function as the preferred habitat.

With the basic building block identified (Fig. 8b), the graph representation of the ID habitat network at Kuching Bay is formulated using the first and second sub-datasets comprising 2 km by 2 km grid cells and the unique individual ID. The former is taken as the distinct location nodes for set L whereas the latter as the distinct dolphin nodes for set D. The data of grid cells in the second sub-dataset that shows visitations by different unique individual ID enables identification of the distinct links for set E. Consequently, thirteen 2 km by 2 km grid cells are identified as the thirteen location nodes, and thirteen unique individual ID identified are taken as the thirteen dolphin nodes of the intended bipartite habitat suitability network (BiHSN). Together with the 38 unique links identified between the bipartite nodes, the complete bipartite graph constructed is defined in Eq. (13) (Liew et al. 2015a p. 269) and presented in Fig. 9.13

**Fig. 9:**
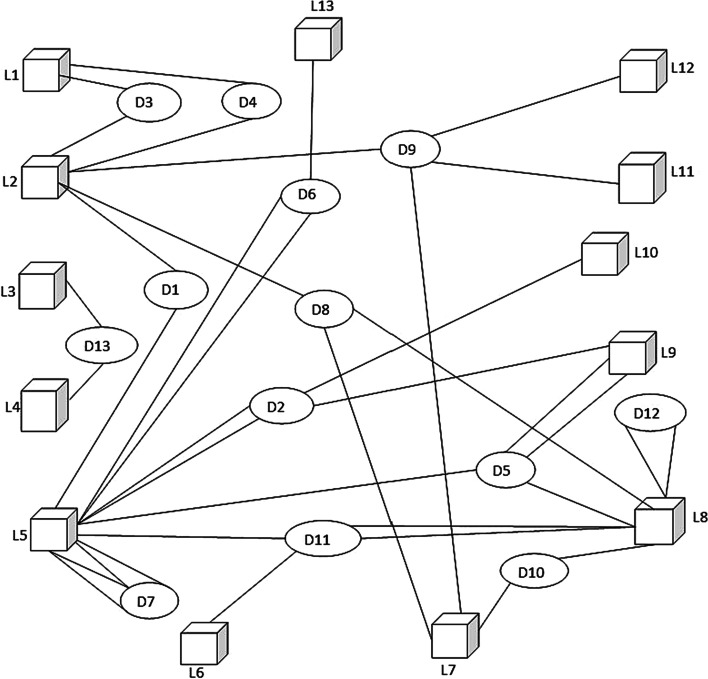
The bipartite habitat suitability graph (Source: Liew 2016, p. 67)

**Fig. 10:**
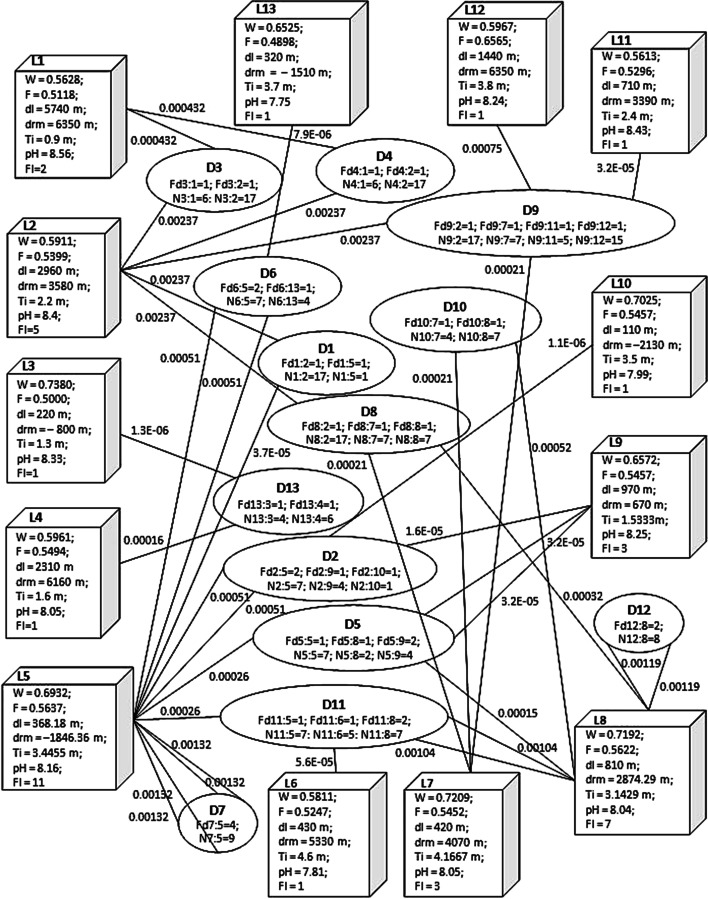
BiHSN with parameter values for location nodes, dolphin nodes and the link weights (Source: Liew et al. 2015a, p. 272)

**Fig. 11:**
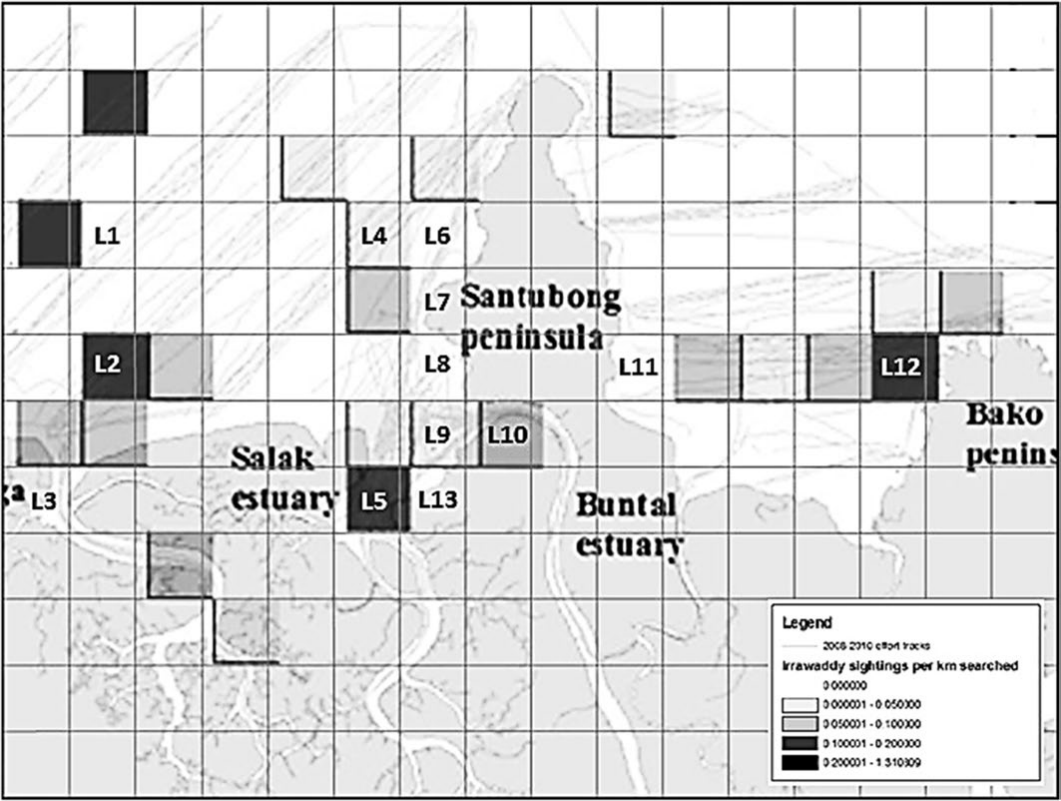
Actual Location Nodes at Kuching Bay (overlaid on modified Fig. 3.5 of Peter (2012)) (Source: Liew et al. 2015a p. 273)

### Bipartite habitat suitability network model construction

In this section, the execution of five important processes of Stage 2 shown in Fig. 1—data pre-processing, location node parameters quantification, dolphin node parameters quantification, link weight quantification, and search algorithm implementation—are discussed.

The data of this study are pre-processed to overcome the missing and faulty values and imbalanced data in the third sub-dataset. The former is achieved through the data interpolation technique (Bavay and Egger 2014) via MATLAB tool for scattered data interpolation on known values of data points. At the same time, the latter is accomplished by applying the under-sampling technique via the systematic random sampling method for Support Vector Machines (SVM), which is the machine learning approach employed in quantifying the location node parameter (Liew et al. 2015c).

The parameters of the location node (node-type-1 in Fig. 1) are determined based on the data available to the study. Twelve parameters are included in this study. They are seawater salinity (*S*) in Practical Salinity Unit (PSU), acidity (*pH*), seawater surface temperature (*T*) in Celsius degree (^o^C), seawater depth (*de*) in meter (m), tide height (*Ti*) in meter (m), water suitability index (*W*), latitude (*x*), longitude (*y*), distance to the river mouth (*drm*) in meter (m) and land (*dl*) in meter (m), fisheries (food) availability index (*F*), and the number of times a location is visited by ID (*Fl*). Out of these twelve parameters, *W*, *F* and *Fl* need to be quantified while the rest are available in the data. The water suitability index (*W*) provides the suitability degree measurement of seawater at a location point of the study location. Conversely, the fisheries (food) availability index (*F*) indicates the availability degree of food for the ID by measuring the possibility of observing fisheries activity at a location point of the study location. This is because the availability of food is a pertinent factor for ID in choosing their preferred habitat. SVM is employed in this study to quantify both *W* and *F* through two distinct machine learning classifiers (Liew et al. 2015a). These two SVM classifiers are SVM Water Model and SVM Fisheries (food) Model formulated through LIBSVM (version 3.17) package (Chang and Lin 2011) with Gaussian radial basis function (RBF) kernel function. The probability estimation of sighting an ID or fisheries activity respectively is extracted from the models and used in this study as the indices. The formal model is defined by six attributes: latitude, longitude, depth, temperature, salinity, and sighting of ID whereas the latter by four attributes: latitude, longitude, tide height, and sighting of fisheries activity.

As for *Fl*, it is given by Eq. 14 (Liew et al. 2015a, p. 270) with *Fd* (dolphin frequency) being a parameter of dolphin node (node-type-2). It intends to rationalize the interaction between the ID and the location through the visitations of ID to a location. Consequently, this study resolves to contain *W*, *F*, *dl*, *drm*, *Ti*, *pH*, and *Fl* as the parameters for the location node where *x*, *y*, *S*, *T* and *de* have already been accounted for in the quantification of *W* and *F*. The values for all the parameters of the location nodes are presented in Fig. 10.14

Likewise, the parameters of the dolphin node (node-type-2 in Fig. 1) are determined based on the data available to the study as well. The two parameters designated for the dolphin node are the number of times a dolphin visited a location (*Fd*) and the best-estimated number of individual ID in the group of ID sighted at a location (N). The former captures the number of times a dolphin node is linked to a location node, as given in Eq. 15 whereas the latter records the group size of each ID sighting as defined in Eq. 16 (Liew et al. 2015a p. 270). Group size refers to the number of individual ID approximated through standard scientific procedures when a group of ID is sighted (Peter 2012). Subsequently, these are the two parameters finalized for this study. The values for these parameters are presented in Fig. 10.1516

For the link formed between any pair of location and dolphin nodes, its weight is referred as HSS. HSS is quantified by incorporating the parameters of both location and dolphin nodes (Liew et al. 2015a). An analysis is carried out on the applicability of different quantification techniques for the link weight, and this study resolved to employ the multiplication rule, as denoted by Eq. 17 (Liew et al. 2015a p. 271). Using this quantification technique, the values of HSS, ranged between zero and one, are computed and presented in Fig. 10, the complete BiHSN.

17 $$\begin{aligned} {\text{HSS}}_{i:j} & = \left( {\prod {\text{Location}}\_{\text{Node}}\_{\text{Parameters}}_{i} } \right) \times \left( {\prod {\text{Dolphin}}\_{\text{Node}}\_{\text{Parameters}}_{j:i} } \right) \\ & = \left( {W_{i} \times F_{i} \times dl_{i} \times drm_{i} \times Ti_{i} \times pH_{i} \times Fl_{i} } \right) \times \left( {Fd_{j:i} \times N_{j:i} } \right) \\ & \quad {\text{where}}\;i \in \left\{ {1, 2, \ldots , 13} \right\}\;{\text{and}}\;j \in \left\{ {1, 2, \ldots , 13} \right\} \\ \end{aligned}$$

With the quantification of the parameters for location and dolphin nodes, and the link that joins them, the preferred habitat of ID at Kuching Bay is then determined by adapting the HITS search algorithm of Eze et al. (2014), as detailed in Algorithm 1. In this study, the power iteration method is implemented with BiHSN as the searching space and the HSS matrix as the input. The output computed is taken as the ranking index, coined as the Habitat Suitability Index (HSI) in this study. It is defined as the suitability degree measurement for the location nodes of BiHSN, valued between zero and one where the higher the value of HSI for a location node the more preferred the location node is to the ID. The findings show that L2 ranked top among the location nodes, implying that it is relatively the most preferred habitat of ID (or the ID hotspot) at Kuching Bay. Table 4 and Fig. 11 present the results (ranking of location nodes, HSI of each location node, and the actual locations at Kuching Bay) obtained in this study. Finally, the bipartite habitat suitability network model for ID at Kuching Bay is formulated.

**Table 4:** Ranking of location nodes (Source: Liew et al. 2015a p. 273)

Ranking	Location Node	HSI
1	L2	1
2	L1	7.3124×10^–2^
3	L12	6.4007×10^–2^
4	L8	4.8837×10^–2^
5	L7	3.5969×10^–2^
6	L5	3.9070×10^–3^
7	L11	2.7536×10^–3^
8	L6	1.0200×10^–4^
9	L9	1.0279×10^–5^
10	L13	5.5695×10^–7^
11	L10	7.8998×10^–8^
12	L4	3.4821×10^–28^
13	L3	2.7196×10^–30^

### Model analysis and evaluation

This last stage consists of three processes: model verification, model validation and network evaluation analysis. The purpose is to ascertain that the model formulated from the previous stage is verified, validated and evaluated.

Benchmark verification using UCINET 6.0 for Window (version 6.498) (Borgatti et al. 2002) is carried out in this study for the BiHSN model. It compares the HSI computed by the BiHSN model and the benchmark system through the computation of RMSE. The resulting value has fulfilled the RMSE threshold value of no greater than 0.05 set in this study. BiHSN is then validated by computing the SRCC between the BiHSN results and two other sets of data: a different set of real data and a past survey result where the validation process is reported in Liew and Labadin (2017). As for the process 3.3, a few extended analyses are executed to further evaluate the capability of the network modeled. The analyses also assess the effect of uncertainty faced when real data is used like uncertainty in data size, in having the unique individual dolphin data, and in having data for certain parameters. Eventually, the ability of BiHSN model to distinguish location nodes where ID is sighted from those that are not sighted and to predict preferred habitat of ID when a new set of data is used is inspected. These analyses have reported encouraging results, supporting the relevance of BiHSN model as an abstraction of the real-world habitat suitability system of ID at Kuching Bay.

## Discussion

The BNM methodology framework is developed to facilitate the modeling activities in studies that intend to use the bipartite network approach. It is a robust framework where the whole modeling effort is captured within three main stages. The specific processes required by each of the stages are explicitly detailed. As demonstrated in the case studies above, once researchers have identified that it is feasible to employ the bipartite network approach, the BNM framework guides the whole modeling processes that follow. The case studies presented above shows that the BNM framework is applicable in both fields of epidemiology in modeling dengue hotspot, and ecology in modeling the preferred habitat of a marine mammal species.

In the first stage that aims to characterize the research problem of a study, the literature reviews, opinions of experts of the field, and research data are the main inputs. These three components have collectively provided a comprehensive understanding of the research scenario (process 1.1) one is surveying. It includes the identification of the potential variables and assumptions for the study. In process 1.2, the graph structure representation of the study is identified. In the above case studies, the authors have recognized the ET and HST that show how the relationship between the entities being surveyed and modeled (the public places and humans, and locations and dolphins) can be represented as discrete objects of two different natures that can be joined by an edge (the dengue contact strength and habitat suitability strength). The graph structure representation is then modified and later evolves into the basic building block for the graph representation of the studies. In process 1.3, the basic building block is used to formulate the complete bipartite graph and its definition using the research data available. The resulting bipartite graphs (Figs. 7 and 9) are the graph structure version of the bipartite networks the studies intend to model.

In the second stage that targets for model construction, data pre-processing is designated as the first process. Getting ready the research data is pertinent for the next four prominent processes in building the intended model. As shown in the case studies discussed above, issues like determining public places, imbalanced and incomplete data are resolved accordingly. The processed data output is then ready to be used in the next three processes (processes 2.2 to 2.4).

The objectives of processes 2.2, 2.3 and 2.4 are to quantify the parameters for the respective bipartite nodes and the link formed between them. The library of quantification techniques attached to them guides the researchers in the need to analyze and determine scientifically on the appropriate method(s) for the intended purpose. In the above case studies, we have shown how parameters for the public place nodes and human nodes, and locations and dolphins’ nodes in the BDC network and BiHSN are quantified by employing the respective traditional mathematical modeling and computational intelligence techniques as presented in "Bipartite dengue contact network model construction" and "Bipartite habitat suitability network model construction". Different methods have also been adopted to quantify the link weight—DCS and HSS—of the studies. The quantification techniques employed are decided and justified based on the results obtained from the scientific analyses or reviews performed. Hence, the choices of quantification techniques in the library depend closely on the review of the studies of the field, the techniques adopted before in research employing the bipartite network approach across the fields, and other emergent novel techniques like the advantages offered by the computational intelligence.

The output of processes 2.2 to 2.4 is the formation of a bipartite network with values for all the parameters of the bipartite nodes and the weights for all the links that are formed between them. The link weights of the network are then expressed in matrix form and input to process 2.5. A search algorithm is implemented in this process onto the link weight matrix with the bipartite network as the searching space. The library of search algorithm technique attached to process 2.5 points the researcher to choose and justify scientifically a method to be employed here. The case studies above resolved to employ the adapted HITS algorithm and produce ranking indices where the ranking of the nodes was based. Ranking indices in the above case studies were taken and termed as DHR value and HSI. The study was probing into utilizing the ranking indices in seeking the hotspot or preferred habitat of concerns. At this point in the methodology process, the intended bipartite network model was constructed. It was termed the BDC Model and BiHSN Model.

As stipulated in "Bipartite network modeling (BNM) framework" section for process 2.5, the ranking index for the other bipartite node (Node-Type-2) can also be generated depending on the aim of the study. A further study has been conducted for the second case study where leadership in species (Irrawaddy dolphin) is surveyed (Liew and Labadin 2018). Using the state-of-the-art of bipartite-network-based approach and employing the BNM framework, promising results have been obtained and validated.

The model formulated in the previous stage needs to go through the model analysis and evaluation, which is the third stage of the BNM methodology framework before it can be accepted as a model by the respective research communities. Processes 3.1 and 3.2 have specified the need to verify or validate, or both, the bipartite network model. These analyses have been carried out accordingly in the case studies presented above where BDC Model and BiHSN Model are verified or validated, or both.

Lastly, the model is further evaluated using appropriate analysis methods (process 3.3) from the library of network evaluation techniques that are attached to it. In the second case study, seven extended evaluations and analyses have been performed: to inspect the relationship between the properties of the model and the result obtained; to evaluate the effect of uncertainty on the performance of the model; and, to evaluate the potential predictive ability of the BiHSN Model. These extended network evaluation analyses implemented on the results produced by the bipartite network model further strengthen the justification of the use of the bipartite-network-based approach in a study as shown in the above study. Apart from that, process 3.3 is the process where the robust complex network analyses could come into further study the statistical properties of interest to the structure and behavior of the network systems formed. Subsequently, the final output of this stage, which is also the output of the study is a verified, validated and evaluated bipartite network model. It is represented as the final result in the BNM framework.

The BNM framework is also employed in modeling the rabies (Chia et al. 2021) and COVID-19 (Hong et al. 2021) transmissions where hotspots or sources of infection for the respective disease are identified using real-world data. On top of this, Hong et al. (2021) managed to determine the ‘super spreader’ (p.132) of the disease and thus allows COVID-19 high-risk groups of people to be identified for better infectious disease management, particularly at the beginning stage of an outbreak. The studies have played a significant role in curbing the deadly rabies and COVID-19 diseases that are contagious. Alongside the applicability of the BNM framework in modeling individual-based network systems, the bipartite network approach is relevant even in solving research problems with scarce or limited data. The bipartite habitat suitability model formulated in the second case study is an example of a solved real-life network system with as few as thirteen nodes. Consequently, the same BNM framework is believed to be applicable in any study where the bipartite network approach is deemed feasible. Besides the principal processes, three libraries of techniques (one for the quantification of nodes parameters and links weights, one for the implementation of search algorithms, and the other one for the evaluations and analyses of the bipartite network model formulated) are included without specifying the actual technique should be employed for the corresponding processes. This implies the uniqueness of potential techniques applicable in a unique research field or domain. As an example, future works employing BNM in disease transmission of epidemiology studies may look into validating the bipartite model constructed with the conventional compartmental models like the Susceptible-Infected-Recovered (SIR) model or any corresponding emergence SIR models.

It shows the emphasis of the BNM framework for keeping the board abreast of updated emergent scientific techniques, and the importance to consider both novel and traditional methods. Grey system theory (Liu et al. 2012) is an example of a newly emerged methodology. It shares many similarities with the BNM approach where both are capable of handling problems with a small sample and limited scientific knowledge of uncertain systems, typical characteristics of the natural world. The possibility of incorporating grey system theory in the bipartite network approach could be an interesting future research recommendation worth looking into. The techniques identified should be scientifically justified within the context of the research field the study confined to. Besides that, the use of BNM methodology in studies with larger data size and in domains other than epidemiology and ecology are greatly desired in the future. These studies may include, but not limited to, surveying the human mobility, materials as vitreous metals, social media, and Web and Internet structure. They are able to strengthen the genericity and scalability of the proposed BNM methodology framework. Subsequently, it is thus suggested that the BNM framework could serve as a generic methodology for a bipartite network approach across research domains and disciplines.

## Conclusions

In this paper, a generic methodology termed the BNM methodology is developed and proposed for the use of the researcher who intends to employ the bipartite network modeling approach with the heterogeneous features of unique individual node of a network system incorporated. The usability of the methodology has been presented in the modeling of the dengue hotspot identification and the habitat suitability of a marine mammal species that capture the features of distinct individual node of the set of bipartite nodes. The BNM framework has the potential to add value to complex network studies especially when the local rules governing the individual vertices are to be considered. This modeling methodology is believed to be feasible and can be readily extended for studies across research fields where the state-of-the-art of bipartite network modeling approach is deemed applicable and appropriate.

## Data Availability

The data used in the current study are available from the corresponding author on reasonable request.

## Abbreviations

BNM: Bipartite Network Modeling
BDC: Bipartite Dengue Contact
BiHSN: Intended Bipartite Habitat Suitability Network
DCS: Dengue contact strength
DHR: Dengue hotspot ranking
ET: Epidemiological triangle
GPS: Global positioning system
HITS: Hypertext induced topic selection
HSI: Habitat suitability index
HSS: Habitat suitability strength
HST: Habitat suitability triangle
ID: Irrawaddy dolphin
RMSE: Root-Mean-Square Error
SDP: Sarawak Dolphin Project
SRCC: Spearman’s Rank Correlation Coefficient
SVM: Support vector machines
